# In Situ Self-Assembly of Nitrogen-Doped 3D Flower-like Hierarchical Porous Carbon and Its Application for Supercapacitors

**DOI:** 10.3390/molecules29112532

**Published:** 2024-05-28

**Authors:** Liqing Qiu, Hangzhong Liu, Chenweijia He, Shuijian He, Li Liu, Qian Zhang

**Affiliations:** 1 Fujian Provincial Key Laboratory of Eco-Industrial Green Technology, College of Ecology and Resources Engineering, Wuyi University, Wuyishan 354300, China; qiulq@wuyiu.edu.cn (L.Q.); liuhz@wuyiu.edu.cn (H.L.); 2 Department of Chemistry and Chemical Engineering, Nanofiber Engineering Center of Jiangxi Province, Jiangxi Normal University, Nanchang 330022, China; 3 Co-Innovation Center of Efficient Processing and Utilization of Forest Resources, International Innovation Center for Forest Chemicals and Materials, Nanjing Forestry University, Nanjing 210037, China; hechenweijia1@163.com; 4 Shanghai Frontiers Science Center of Advanced Textiles, College of Textiles, Donghua University, Shanghai 201620, China; liliull@dhu.edu.cn

**Keywords:** flower-like porous carbon, hydrothermal method, starch, supercapacitor

## Abstract

The hierarchical porous carbon-based materials derived from biomass are beneficial for the enhancement of electrochemical performances in supercapacitors. Herein, we report the fabrication of nitrogen-doped 3D flower-like hierarchical porous carbon (NPC) assembled by nanosheets using a mixture of urea, ZnCl_2_, and starch via a low-temperature hydrothermal reaction and high-temperature carbonization process. As a consequence, the optimized mass ratio for the mixture is 2:2:2 and the temperature is 700 °C. The NPC structures are capable of electron transport and ion diffusion owing to their high specific surface area (1498.4 m^2^ g^−1^) and rich heteroatoms. Thereby, the resultant NPC electrodes display excellent capacitive performance, with a high specific capacitance of 249.7 F g^−1^ at 1.0 A g^−1^ and good cycling stability. Remarkably, this implies a superior energy density of 42.98 Wh kg^−1^ with a power density of 7500 W kg^−1^ in organic electrolyte for the symmetrical supercapacitor. This result verifies the good performance of as-synthesized carbon materials in capacitive energy storage applications, which is inseparable from the hierarchical porous features of the materials.

## 1. Introduction

Nowadays, various electrochemical energy storage devices have been developed based on versatile nanomaterials and nanotechnologies [1,2,3]. Supercapacitors (SCs), as advanced energy storage devices, have drawn widespread attention owing to their fast charge–discharge rates, efficiency-added energy conversion, high power density, long cycling life, environmental friendliness, etc. [4,5,6,7]. A typical SC is composed of two electrodes, an electrolyte, a separator, and two current collectors. In line with the performance of the SC, enhancement of energy density (E) without the depletion of power density (P) should be the goal, as achieving this will contribute to the development of an efficient energy conversion and storage system [8,9]. This is based on the equation of E, which shows a positive correlation with capacitance and even the square of the voltage range for E [10,11]. As is known to us, the electrode materials act as the core element for improving capacitance. In particular, there exist metal oxides [12], conducting polymers [13], Mxenes [14,15], carbons [16], etc., which are applied as workable electrodes. Biomass-based precursors, such as rape pollen [9], lignin [17], durian rind [18], waste paper [19], and wood [17,20], are widely used in preparing electrodes, adsorbents, etc. Biomass is rich in carbon elements and widely converted into carbon materials [21,22,23]. Thanks to its low cost, renewability, stability, and non-toxicity, a growing number of biomass sources are being introduced into this field. In line with this, it makes sense to choose starch (a biomass-derived product) as the role to produce a fascinating carbon.

Starch, as a natural polysaccharide, possesses a high carbon content, making it a promising candidate for conversion into high-value carbon materials. At present, carbon aerogels, carbon foams, carbon microspheres, etc., derived from starch-derived raw materials demonstrate excellent performance in supercapacitors [24,25,26,27,28]. As can be noted, the common pursuit outlined above is the hierarchical porous structure, involving micro/meso/macro-pores, especially mesopores. The advantages can be integrated in terms of overall electroactive sites, abundant charge storage, fast ion diffusion, and rapid electron transfer, resulting in actual capacitance, rate capability, and cycling stability [29]. However, the expensive template, toxic (corrosive) reagent, as well as catalyst, is exploited during (after) the carbonization [28,30,31]. Additionally, introducing rich heteroatom doping could render a further improvement to the electrochemical properties of carbon-based electrodes [32]. Hence, it is essential to develop a simple and effective approach for harvesting useful carbon materials.

To date, the hydrothermal method, as a rising bio-synthetic strategy, has achieved various porous carbons, such as porous carbon spheres and carbon sheets, etc. [33,34]. This work inspired us to use hydrothermal technology to prepare 3D flower-like porous carbon from starch. The existence of this substance could shorten the space between electrolyte and electrode materials, resulting in higher efficiency in charge storage and transmission. Generally, a surfactant or structural inducer is needed to facilitate the process of growth [24,35]. In other aspects, the introduction of heteroatoms through mono/dual/multi-doping alters the electron mobility within the carbon bulk, while also improving the wettability of the matrix and electrolyte. This enhancement results in improved electrochemical performance for carbon electrodes. Shan et al. achieved co-doped (N and S) porous carbon spheres by mixing cellulose and ammonium sulfate solution via hydrothermal carbonization at 300 °C [36]. Xu et al. used the incorporation of ZnCl_2_ and urea to obtain organic matter from sewage sludge at 210 °C through hydrothermal techniques [37]. However, these methods suffer from dealing with insecure high temperatures. Herein, both urea and ZnCl_2_ are dispersed in deionized water during the low hydrothermal reaction, acting as a heteroatom source and activated template, respectively.

In this work, we achieve hierarchical nitrogen-doped 3D flower-like porous carbon by hydrothermal and carbonization means, formed by the combination of starch, urea, and ZnCl_2_. We optimize the effects on the morphology, structure, and properties, which are regulated by the mass ratio of the mixtures and carbonized temperature. Owing to the morphology of the NPC architecture with a high specific surface area (1498.4 m^2^ g^−1^) and heteroatom doping, the optimized carbons enable great electrochemical performance for SC, such as capacitance, rate performance, and cycling stability in both KOH and TEABF_4_/AN electrolytes.

## 2. Results and Discussion

### 2.1. Formation of NPCs

**Scheme 1:**
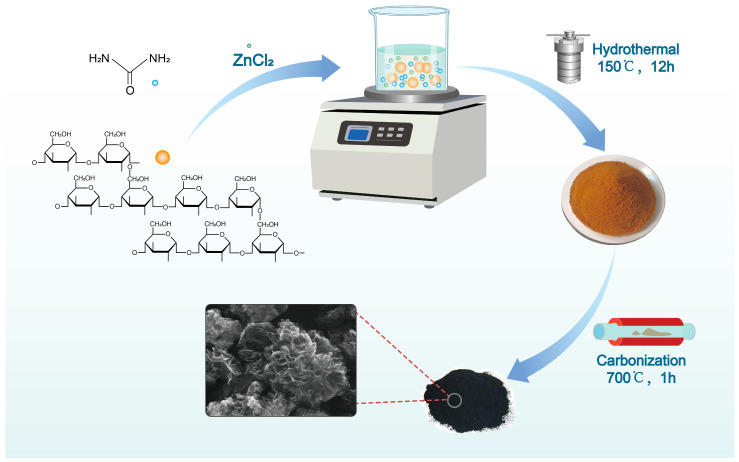
The synthesis route of nitrogen-doped 3D flower-like hierarchical porous carbon material from starch.

The synthesis route is shown in Scheme 1. The mixture containing starch, urea, ZnCl_2,_ and deionized water was transferred into a 100 mL stainless steel autoclave to obtain the pre-carbonized brown resultant, which then underwent the targeted carbonization process. Urea and ZnCl_2_ were used as a dopant and an activator, respectively. On the one hand, hydroxy dichlorozincic acid and zinc oxide were formed from the reaction of ZnCl_2_ and H_2_O, which played a crucial role as an etching activator and template leading to flower-like porous structures [38,39]. On the other hand, the mass ratio of urea and ZnCl_2_ had a deep influence on the porous structure and nitrogen doping, which affected the final electrochemical performance.

### 2.2. Morphology of NPCs

**Figure 1:**
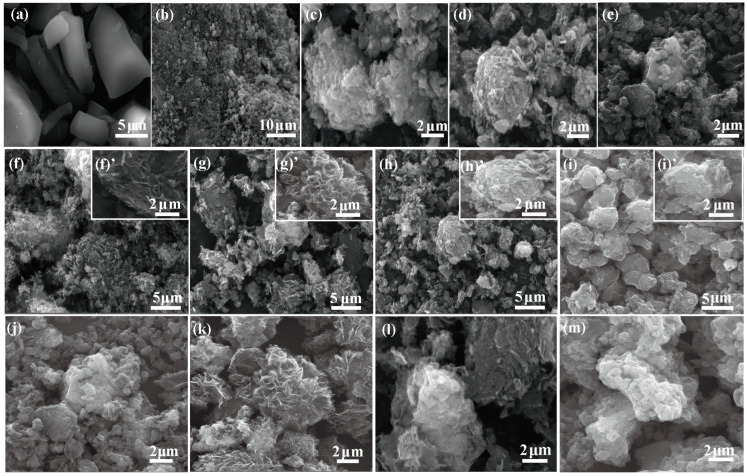
SEM images of NPC samples (**a**–**e**) named Pure Starch, CS-700, NPC-700-3.5-1, NPC-700-3.5-2, and NPC-700-3.5-3, respectively; (**f**–**i**) named NPC-700-1-2, NPC-700-2-2, NPC-700-3.5-2, and NPC-700-4-2, as well as its enlarged images shown in (**f’**–**i’**), respectively; (**j**–**m**) named as NPC-600-2-2, NPC-700-2-2, NPC-800-2-2, and NPC-900-2-2, respectively.

Scanning electron microscopy (SEM) revealed the microstructure of the obtained samples. It was reported that plenty of well-organized microstructures developed after carbonization, apart from those found in natural starch and directed grains. In contrast, the original materials exhibit a typical brick-like stone structure with no obvious pores, whereas the stones were crushed into ordinary particles on the carbonaceous surface after hydrothermal treatment and carbonization of CS-700 (Figure 1a,b). It is worth mentioning that several types of porous carbons were fabricated, including sheet-like and flower-like porous carbon, under different tests for other products (Figure 1c–m). Herein, the carbon microstructures from the growth of carbon sheets in an overall direction with the size of particles were approximately 5 µm (inserted in Figure 1f’–i’), which is derived from such high-pressure treatment via facile hydrothermal strategy [24]. As we know, a joint structure combining flower-like geometry with interconnected pore channels can render fast ion diffusion paths to reduce the electric resistance, which is in agreement with the specific surface areas and pore volumes discussed above [40]. As shown in Figure 1c–e, structures with reasonable ZnCl_2_ concentrations may have pore-accessible distribution, leading to satisfactory capacitive behaviors. It made sense that the morphology appearance varied when the urea/ZnCl_2_ ratio was changing, demonstrating that further pyrolysis of different urea/ZnCl_2_ mass ratios was indeed needed. In the case of the temperature element, as the temperature increased, a perfect flower-like structure emerged along with interconnected pores. However, it led to drastic shrinkage during carbonization, resulting in a collapsed structure with the increasing temperature, as seen in Figure 1l,m. Hence, the carbonization temperature was a significant key factor that affected the properties of a series of NPC samples. 

**Figure 2:**
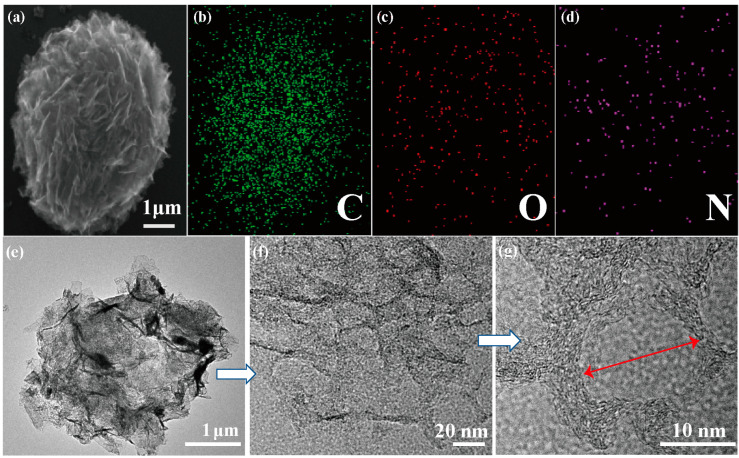
The NPC-700-2-2 sample: Elemental mappings (**a**–**d**); TEM images and its High-resolution morphology (**e**–**g**).

To further clarify the structure of the NPC-700-2-2 sample, TEM images were obtained subsequently, which are portrayed in Figure 2e–g. In detail, the TEM images showed a very similar ordered structure compared with the SEM image in Figure 1g. Microscopically, it was evident that there was almost no long-range order by way of enlargement, which attested to the amorphous structure, corresponding to the analysis of following the X-ray diffraction (XRD) pattern and Raman spectra. Figure 2g (marked with a red double arrow) indicates the formed mesoporous due to the template originating from ZnO particles. According to the results presented in Figure 2a–d, C, N, and O were indeed well-prepared, highlighting the excellent nitrogen doping process.

### 2.3. Phase Structure and Surface Chemistry Characterization

**Figure 3:**
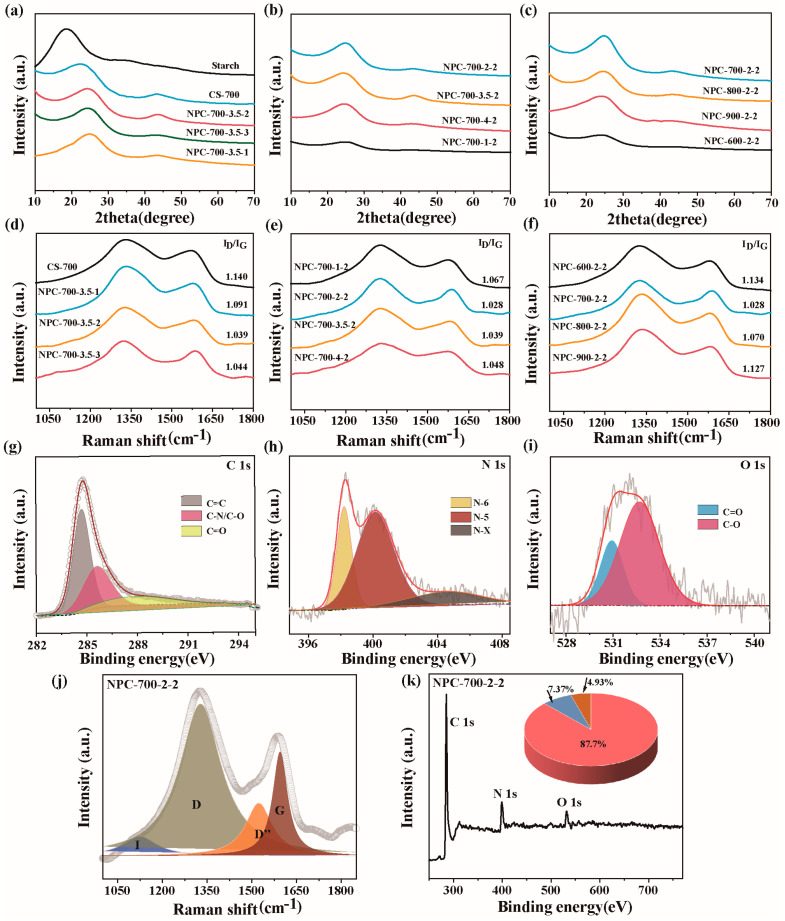
XRD pattern (**a**–**c**); Raman spectra (**d**–**f**) of NPCs; the NPC-700-2-2 sample: the high-resolution XPS spectra of C 1s, N 1s, and O 1s (**g**–**i**), fitting Raman spectra (**j**), and XPS survey spectrum (**k**) of NPC-700-2-2.

Figure 3a–f displayed a representative X-ray diffraction (XRD) pattern and Raman spectra of pristine starch, CS-700, and the NPCs. As shown in Figure 3a–c, compared with a single peak at 18° for pristine starch, the others exhibited two diffraction peaks located at about 25° and 44°, corresponding to typical (002) and (101) crystal planes of graphite carbon, respectively. This can be ascribed to the existence of structural strain [41,42]. Moreover, the optimal mass ratio was 2:2 between urea and ZnCl_2_, as explained by adsorption energy (Eads) [43]. In general, the more negative the value of Eads, the more stable the structure was, resulting in more graphitization grains (see Figure 3a,b). To some extent, a preferable degree of graphitization occurred with the improvement of charring temperature, as shown in Figure 2c. The coexistence of the D band and the G band were two characteristic peaks related to disordered carbon and graphitic carbon, which were calculated by area ratio, respectively (Figure 3d–f). The values of the I_D_/I_G_ ratio in the Raman spectra represented the degree of graphitization, which was not related to defects directly. The I_D_/I_G_ values for a series of NPC samples were 1.091, 1.039, and 1.044, whereas the values were lower than the CS-700 sample (1.140), implying a higher graphitization degree of NPCs. Furthermore, the value initially increased and then decreased as the temperature rose continuously, resulting in the lowest I_D_/I_G_ value of 1.028. Distinctly, compared with the temperature element, there was only a slight change in the I_D_/I_G_ intensity ratio with variation of urea and ZnCl_2_. Hence, rational nitrogen doping (derived from urea) and pores (derived from ZnCl_2_) result in an enhanced degree of graphitization, which is in agreement with the XRD results. Furthermore, four peaks located at the I band (1121 cm^−1^), D band (1327 cm^−1^), D″ band (1524 cm^−1^), and G band (1597 cm^−1^) were observed after the fitting operation, as described in Figure 3j. The results showed the degree of graphitization and defect for the D, D″, and G bands. Meanwhile, the I band showed the linkage between heteroatom (N) into the carbon walls, corresponding to XPS and EDS [44,45]. 

Furthermore, the surface chemistry characteristics of the accurate content of each element as well as the chemical bond type were measured via XPS. As depicted in Figure 3k, the survey of NPC-700-2-2 had three peaks at 284.5, 398.8, and 531.8 eV that were indexed as C 1s, N 1s, and O 1s, demonstrating the incorporation of N and O into the carbon matrix, along with doping content of 7.37 and 4.93 at%, respectively. The high-resolution spectra of C 1s were split into three peaks, located at ~284.7, ~285.59, and ~287.97 eV, which corresponded to C=C, C-N (C-O), and C=O (Figure 3g). As shown in Figure 3i, two typical divided peaks in the O 1s spectrum centered at 530.92 and 532.68 eV represented the C=O and C-O bonded chemical state. For the fitting results of N 1s, three individual peaks emerged at 398.2, 400.11, and 404.65 eV, corresponding to pyridinic nitrogen (N-6), pyrrolic nitrogen (N-5), and pyridine-N-oxide (N-X), further providing evidence for N-doping embedded into carbon frameworks [46]. Therefore, the presence of N-doped or oxygen-containing bonded carbon atoms can not only enhance their electrochemical performance but also facilitate the wettability between the interface of the electrode and the electrolyte, leading to a reduction in the internal resistance by way of the formation of polar functionalities [47].

### 2.4. Textural Structure

**Figure 4:**
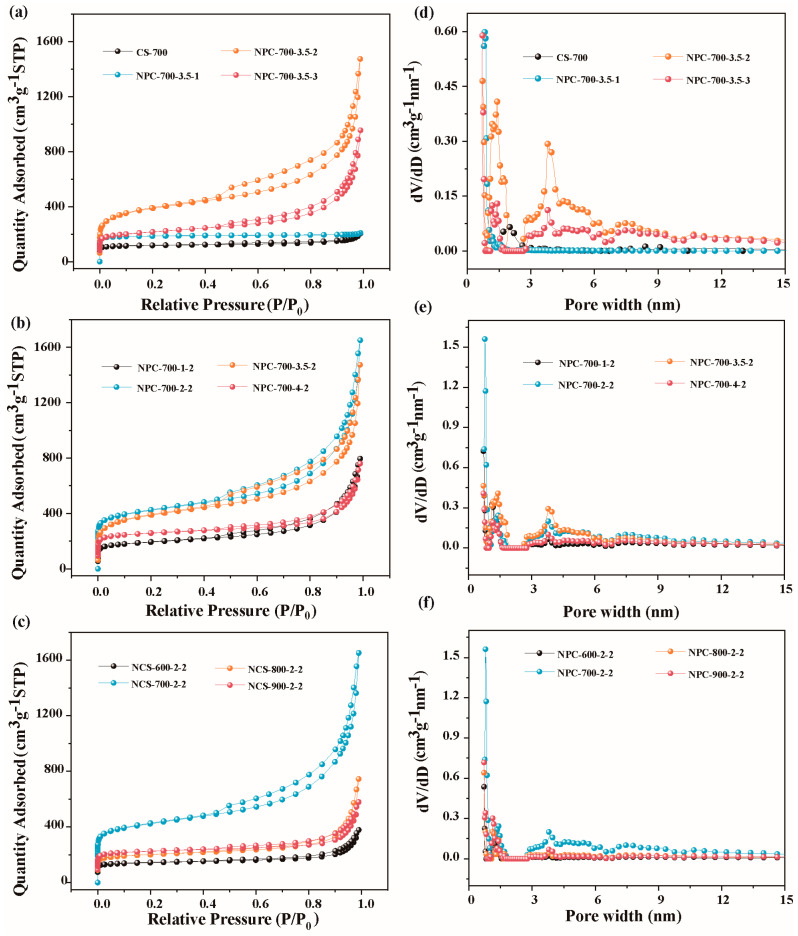
(**a**–**c**) Nitrogen adsorption/desorption isotherms; (**d**–**f**) PSD curves of NPCs.

The nitrogen adsorption–desorption isotherms and pore size distribution (PSD) of the materials after pyrolysis are depicted in Figure 4, and the corresponding pore structure parameters are shown in Table 1. Usually, the error parameters for BET are about 2–5%, which is acceptable. As shown in the picture, in a relatively low-pressure region (P/P_0_ < 0.05), all the samples demonstrated the formation of large amounts of micropores after carbonization. Within a moderate relative pressure range (P/P_0_ = 0.45–0.9), a scene of an H_4_-type hysteresis loop formed for other products (type IV isotherms) compared with both CS-700 and NPC-700-3.5-1 materials (type I isotherms), implying the presence of meso-porosity. At the relatively high-pressure region (P/P_0_ > 0.9), a sharp increase in adsorption capacity occurred due to the presence of macropores [40]. It was known that type I isotherms possessed pore sizes mainly less than 2 nm, as calculated by the DFT model, which is in accordance with the PSD curves and the average pore size (D_aver_) portrayed in Figure 4d and Table 1. On the contrary, type IV isotherms had porous carbons with different pore sizes, which is favorable for electron and ion diffusion, thereby endowing enhanced electrochemical performance [48].

As the mass ratio of urea to ZnCl_2_ increased from 3.5:1 to 3.5:2, the specific surface area of the sample increased from 450 to 1178 m^2^ g^−1^. Further extending the mass ratio to 3.5/3 resulted in a decrease in specific surface area to 779 m^2^ g^−1^, indicating that the contribution of micropores exceeded 96% within the specific surface area, which was also consistent with the structure of Raman analysis results after ZnCl_2_ overuse. With the increasing urea content since ZnCl_2_ was fixed, we can reach a maximum surface area of 1498 m^2^ g^−1^ (NPC-700-2-2) in four materials, along with those values ascending first and then decreasing. Finally, in the case of temperature variation, the identical trend illustrated in the specific surface area, as mentioned above, was mainly due to the collapse of pore structures at different temperatures. The pore size distribution of the samples was regulated by pictures and data, respectively. In a word, the porosity of the as-prepared samples can be assigned to the ZnCl_2_ activation and interaction between the urea and ZnCl_2_. The processes of dehydration, release of H_2_O and CO_2_, etc., and washing to remove the remaining ZnCl_2_ from the carbon precursors could enrich the porosity. Therefore, besides the large surface area, porous structures with various pore sizes were beneficial for fulfilling their excellent capacitive behavior [40].

### 2.5. Electrochemical Performance

**Figure 5:**
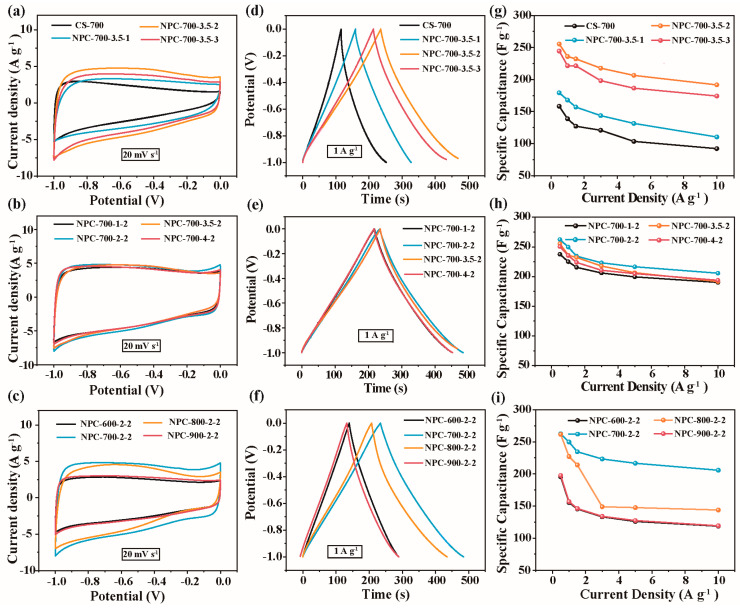
Electrochemical characterization in a three-electrode system of NPCs: (**a**–**c**) CV curves tested at 20 mV s^−1^; (**d**–**f**) GCD curves measured at 1 A g^−1^ and (**g**–**i**) specific capacitance at different current densities.

**Figure 6:**
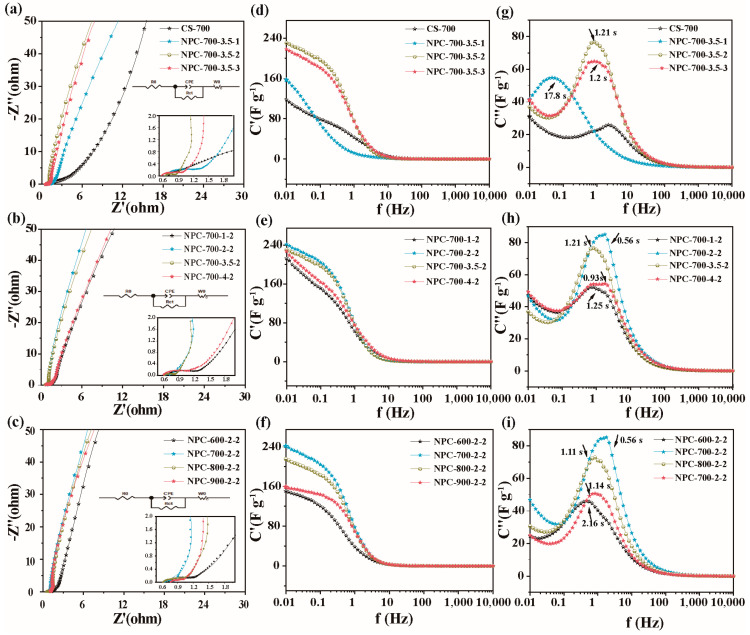
Nyquist plot curves (**a**–**c**); the real capacitance (**d**–**f**); progression of the imaginary capacitance (**g**–**i**) of NPCs.

Cyclic voltammetry (CV) and galvanostatic charge/discharge (GCD) were measured in a three-electrode configuration. The CV plots were portrayed in Figure 5a–c at 20 mV s^−1^, exhibiting the near-rectangle curves that could respond to the electrical double-layer capacitance behavior. It is worth mentioning that the NPC-700-2-2 electrode, which was assembled with a 2:2 mass ratio of urea to ZnCl_2_ and 700 °C of carbonization, was composed of the best-enclosed region area in contrast to those under other conditions. In addition, samples of the GCD curves at 1 A g^−1^ depicted in Figure 6d–f showed symmetrical curves, the tendency for which was consistent with the CV curves. The largest specific capacitance was observed in either the embraced area of CV or the discharge time of GCD, corresponding to well-ordered pore structures and good conductivity. Unexpectedly, the mass ratios of urea to ZnCl_2_ were 1:2 (2:2, 3.5:2, 4:2), which had a litter effect on the electrochemical capacitance. Interestingly, the calculated specific capacitances of the electrodes from the discharge time in GCD curves dropped gradually at 0.5, 1, 1.5, 3, 5, and 10 A g^−1^, respectively. As mentioned above, it can be suggested that it was difficult to achieve electron and ion transport at a larger current density. Additionally, the Cs (specific capacitances) showed an optimal scene when compared with those results obtained from biomass-derived precursors previously, as seen in Table 2.

Electrochemical impedance spectroscopy (EIS) was conducted to study the kinetic behavior of electrode materials during the electrochemical reaction process [54,55]. It was carried out at an open-circuit potential (OCP) of 0.0 V and a frequency ranging from 0.01 Hz to 100 kHz. The inset figure was the fitted equivalent circuit of as-obtained electrodes [38]. EIS Nyquist plots and the corresponding fitting curves were obtained, including the actual and virtual capacitance in terms of frequency. According to frequency range, two sections were divided as seen in Figure 6a–c, with vibrio-shaped and oblique lines at the high and low frequency regions, respectively. In the high-frequency region located by the X-intercept, the smallest intrinsic ohmic resistance (R_0_ = 0.63 Ω) of NPC-700-2-2 was obtained, proving that it had the best conductivity. Meanwhile, a smaller semicircle radius indicates a lower charge-transfer resistance between the active material and electrolyte. The low-frequency region was attributed to the oblique line, meaning that the nearly vertical plots of the as-prepared electrodes indicated an efficient electrolyte diffusion rate, benefiting from the dominant porous texture [56]. In addition, the actual capacitance (C′) was the effective capacitance that the material can afford, determining the values of the specific capacitance. Based on the corresponding analysis as Figure 6d–f, it depended on a frequency strong shift from 0.01 Hz to 5 Hz, and subsequently arrived nearly equal to the constant when the frequency exceeded 5 Hz. Notably, the highest initial capacitance was approaching the calculated data (the result of CS-700 was clearly different and lower compared to the others), which is in agreement with the diagram in Figure 5. The virtual capacitor (C″) looked like a parabola with an upward opening, reaching its maximum value at a frequency ƒ_0_. A time constant was defined as τ_0_ = 1/ƒ_0,_ aiming to reflect the reversibility of the charge-discharge progress of the electrodes. The τ_0_ of NPC-700-2-2 was 0.56 s, which was much smaller than others (Figure 6g–i), indicating that it had relatively good capacitance performance. 

**Figure 7:**
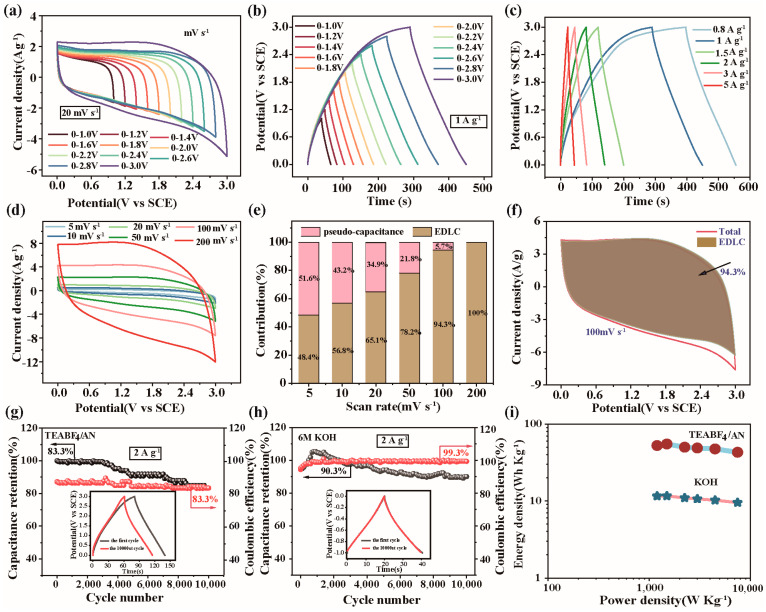
The NPC-700-2-2 sample in the two-electrode system with TEABF_4_/AN electrolyte: (**a**) CV curves under different voltage windows at the scan rate of 20 mV s^−1^; (**b**) GCD curves in different operation voltages at the constant density of 1 A g^−1^; (**c**) GCD curves at different current densities; (**d**) CV curves at different scan rates; (**e**,**f**) pseudo-capacitance and EDLC contributions at various scan rates in CV curves at 100 mV s^−1^, reflecting the pseudo-capacitance contribution; (**g**) cycling stability of NPC-700-2-2//NPC-700-2-2 tested at 2 A g^−1^ for 10,000 cycles, including GCD curves for the first and 10,000th cycles; (**h**) cycling stability of NPC-700-2-2//NPC-700-2-2 measured at 2 A g^−1^ for 10,000 cycles with 6 M KOH, and its first and final cycles; (**i**) diagrams of power density versus energy density under 1 M TEABF_4_/AN and 6 M KOH electrolytes.

An alkaline electrolyte (6 M KOH) was not optimal due to its low voltage window. Therefore, an IL electrolyte (TEABF_4_/AN) was utilized to broaden its potential voltage range on account of its adjusting ability, as portrayed in Figure 7. Figure 7a,b depicts CV curves (at 20 mV s^−1^) and GCD curves under a variety of windows (at 1 A g^−1^), signifying that a wide working voltage can reach up to 3 V, and the CV curve shape retained the similarity gradually with the growing windows, which was consistent with a synchronous charge–discharge process. Macroscopically, CV curves and GCD curves at various scan rates and current densities under 0–3 V were further studied. The results are shown in Figure 7c,d. Unexpectedly, quasi-rectangular CV curves shifted to the tilted region, which can be ascribed to high interference impedance under 200 mV s^−1^ of high scan rate accompanied by sluggish ion transfer via micropores. There were two formal behaviors, including pseudo-capacitance and EDLC. The contribution of the capacitance-controlled process increased with the scanning rate, reaching 48.4, 58.6, 65.1, 78.2, 94.3, and 100% at scan rates of 5, 10, 20, 50, 100, and 200 mV s^−1^, respectively (Figure 7e,f). The cycling stability (10,000 cycles) of supercapacitors based on the NPC-700-2-2 sample under two series electrolytes was tested (at 2 A g^−1^) in Figure 7g,h. It was obvious that the capacitance retention rate of the IL electrolytes, which was 83.3%, slightly preceded that of the alkaline electrolytes, which was 90.3%. However, the results of the coulombic efficiency were contrary to expectations. As anticipated, the Ragone curve of power density versus energy density in IL electrolytes was excellent in comparison to that in the KOH electrolytes. For the NPC-700-2-2 electrode in the IL electrolytes, the maximum energy density was 52.63 Wh kg^−1^, corresponding to 1200 W kg^−1^ of the power density. Meanwhile, even with a further increase in power density to 7500 W kg^−1^ in 1 L electrolytes, the available energy density still remained at 42.98 Wh/kg^−1^. Hence, this finding provided a promising route to obtain an attractive electrode material for use in high-energy-density supercapacitors.

### 2.6. Mechanisms of Energy Storage and the Synergistic Effect

**Figure 8:**
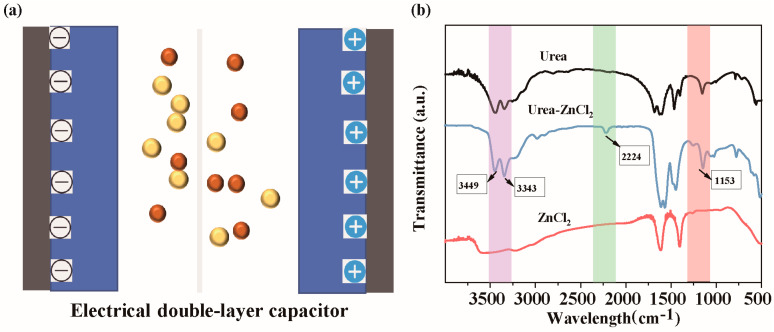
Illustration of EDLC (**a**); FTIR spectra of urea, urea-ZnCl_2,_ and ZnCl_2_, respectively (**b**).

Based on the analysis above, the energy storage focused on the typical EDLC and pseudo-capacitance. Rectangular-like curves derived from cyclic voltammetry exhibited the representative behaviors (EDLC), which stored and released charges through the electrical double-layer interface between electrode and electrolyte, accompanied by the adsorption or desorption process, shown in Figure 8a. In addition, the pseudo-capacitance originated from the faradic redox reactions owing to the rich nitrogen content (N-6, N-5, and N-X, shown in Figure 3), as the following Equations (1) and (2) [57]. Herein, the nitrogen doping and hierarchical porous structure had a synergistic effect on the efficient energy storage. As is known, urea and ZnCl_2_ acted as the dopant and activator, respectively. Firstly, the characteristics of the low melting point of ZnCl_2_ and small Zn^2+^ size boosted the formation of nanopores during hydrothermal carbonization. Furthermore, the interaction between ZnCl_2_ and H_2_O created hydroxy dichlorozincic acid and zinc oxide, which played a role as an activator and template [39,58,59]. Secondly, a new peak at 2224 cm^−1^ indicated the interaction between urea and ZnCl_2_ (the FTIR spectra in Figure 8b), which caused the increase in nitrogen in the carbon wall owing to the existence of a more stable structure.

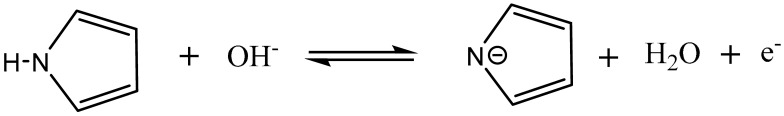
(1)

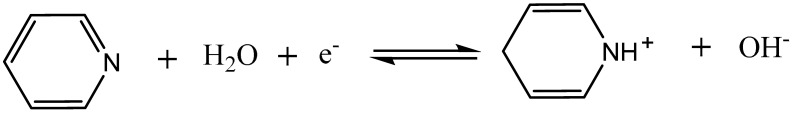
(2)

## 3. Experimental Section

### 3.1. Synthesis of Starch-Derived Porous Carbon

All the chemicals and reagents used were analytical grade. Typically, a mixture of starch (S, 2 g), urea (U, 2 g), and ZnCl_2_ (Z, 2 g) was dispersed in 50 mL of deionized water and the mixture was stirred mechanically for 1 h. Afterward, they were poured into an autoclave reactor with a volume of 100 mL and kept at 150 °C for 12 h. After cooling to room temperature, the samples were dried after filtration. Then, the prepared resultants above were heated to different temperatures with a heating rate of 5 °C/min in an N_2_ flow (e.g., 600, 700, 800, and 900 °C) and held for 1 h. Finally, they were alternatively soaked in dilute HCl and H_2_O using ultrasonication until neutral pH was achieved, ensuring the absence of Cl^−^. The final samples were denoted as NPC-700-2-2, where 700 refers to the carbonization temperature, and 2 and 2 refer to the mass of U and Z, respectively. A series of mass ratios of S to U and Z was set as 2:3.5:1, 2:3.5:2, 2:3.5:3, 2:4:2, 2:2:2, and 2:1:2 to explore the material ratios for the prepared samples. Hence, they were labeled as NPC-700-3.5-1, NPC-700-3.5-2, NPC-700-3.5-3, NPC-700-4-2, and NPC-700-1-2 by the same method, respectively. For comparison, starch alone was placed in 50 mL of H_2_O and placed in a 100 mL autoclave reactor for hydrothermal reaction and carbonization (named CS-700). Note that the NPC had characteristics of low density and fluffiness, in comparison with the acquainted active carbon (AC) and carbon black (CB). 

### 3.2. Characterization and Electrochemical Measurement

The crystalline structure was investigated by X-ray diffractometer (XRD, D8 ADVANCE, Bruker, Billerica, MA, USA) with Cu Kα radiation (λ = 1.54056 Å) and Raman Spectroscopy (Raman, Alpha 300WITec, Oxford Instruments, Abingdon, UK) with a He-Ne laser beam equipped with an excitation wavelength of 532 nm [60]. The pore textures were measured based on N_2_ adsorption/desorption isotherms using an autosorb-iQ analyzer at 77 K after degassing at 200 °C for 10 h. Values of SSAs and the total pore volumes were calculated using the Brunauer–Emmett–Teller (BET) method and the nitrogen volumes adsorbed at the maximum relative pressures equal to 0.99, respectively. The pore size distribution curves were derived from the density functional theory (DFT) model, assuming slit pore geometry. The morphology and surface elemental distribution were observed by a scanning electron microscope (SEM, VEGA3, TESCAN, Brno, Czechia) which carried an energy-dispersive X-ray spectrometer (EDS, ULTIMMAX, Oxford Instruments, Abingdon, UK). The microstructure was investigated using a transmission electron microscope (TEM, JEM2100, JEOL Ltd., Tokyo, Japan). The surface elements and chemical valence states of the specific sample were tested by the X-ray photoelectron spectra (XPS, AXIS SUPRA, Shimadzu, Kyoto, Japan) with Al-Kα radiation of 1253.6 eV. The step sizes of full survey spectra (pass energy of 150 eV) and the high-resolution spectra (pass energy of 50 eV) were 1 eV and 0.1 eV, respectively.

The electrochemical properties of the NPC electrodes, which contained cyclic voltammetry (CV) curves, galvanostatic charge/discharge (GCD) curves, and electrochemical impedance spectra (EIS) (0.01 Hz to 100 kHz), were investigated by an electrochemical workstation (CH1760E, Shanghai Chenhua Instrument Co. Ltd., Shanghai, China). The calculated details of specific capacitance, energy density, and power density were consistent with ref. [40]. The working electrodes were prepared by mixing as-obtained NPC (about 2.5–4 mg mass loading), acetylene black, and polytetrafluoroethylene with a mass ratio of 8:1:1. Afterward, it was ground with adequate N-methyl pyrrolidone. Finally, it was painted and pressed upon the Ni foam at the pressure of 10 MPa for 1 min. The capacitive performance of porous carbon materials was evaluated in a three-electrode setup and two-electrode setup. In the three-electrode setup, 6.0 mol/L KOH solution served as the electrolyte, while the reference electrode and the counter electrode were a saturated mercuric oxide electrode and a platinum sheet electrode, respectively. In a symmetric system, 1.0 M TEABF_4_/AN solution was used as the electrolyte, and two identical electrodes with the same mass loading were used as the cathode and anode.

## 4. Conclusions

In this paper, hierarchically nitrogen-doped 3D flower-like porous carbon was fabricated through the addition of starch, urea, ZnCl_2_, and deionized water via a low-temperature hydrothermal reaction and carbonization process. By structural characterization, including XRD, Raman, SEM, XPS, and TEM, as well as specific surface areas and pore volumes, the parameters for mass ratio and temperature were selected. The results showed that the biggest specific capacitance can reach 249.7 F g^−1^. Furthermore, the fabricated symmetrical capacitor displayed a high energy density of 42.98 Wh kg^−1^ at a power density of 7500 W kg^−1^. Therefore, the facile progress for both hydrothermal methodology and carbonization at moderate temperatures showed great potential for more precursors of interesting porous structures, which are beneficial for large-scale applications due to their good electrochemical performance as carbon-based electrodes.

## Figures and Tables

**Table 1:** Specific surface areas and pore volumes of NPCs.

Sample	Specific Surface Area (m^2^ g^−1^)		Pore Volume (cm^3^ g^−1^)		
	S_BET_	S_micro_	V_total_	V_micro_	D_aver_ (nm)
CS-700	332	302	0.22	0.11	1.0
NPC-700-3.5-1	450	432	0.25	0.14	2.0
NPC-700-3.5-2	1178	839	1.69	0.38	7.8
NPC-700-3.5-3	779	775	0.75	0.23	6.7
NPC-700-1-2	695	531	0.95	0.19	7.3
NPC-700-2-2	1498	1163	1.93	0.44	6.9
NPC-700-4-2	1009	893	0.88	0.31	5.2
NPC-600-2-2	637	589	0.44	0.18	4.7
NPC-800-2-2	934	859	0.68	0.29	6.5
NPC-900-2-2	839	742	0.76	0.24	4.5

**Table 2:** The summary of specific capacitance from different materials.

Electrode Material	Activator/Template	Cs (F g^−1^)	Electrolyte	Ref
Carbon microspheres (starch)	CTAB	242.5 (1 A g^−1^)	6 M KOH	[24]
Microspheres (cellulose)	Ammonium Sulfate	227.3 (1 A g^−1^)	6 M KOH	[36]
Nanoporous carbon (PF resin)	Urea/ZnCl_2_	204 (1 A g^−1^)	6 M KOH	[43]
NCs (tyrosine)	Urea/ZnCl_2_	235.8 (1 A g^−1^)	6 M KOH	[40]
Xerogel (tyrosine)	KOH	302 (0.5 A g^−1^)	6 M KOH	[47]
HPC (starch)	Mg (NO_3_)_2_	229 (1 A g^−1^)	6 M KOH	[49]
Activated Carbon (sugar)	H_2_SO_4_/KOH	242.67 (1 A g^−1^)	6 M KOH	[50]
Porous carbon (lotus)	KOH	214.5 (1 A g^−1^)	6 M KOH	[51]
Porous carbon (biomass waste)	CaCO_3_	232.8 (1 A g^−1^)	2 M KOH	[52]
Porous carbon (ulva lactuca)	Ni catalyst	167 (1 A g^−1^)	6 M KOH	[53]
NPCs (starch)	Urea/ZnCl_2_	249.7 (1 A g^−1^)	6 M KOH	This work

## Data Availability

The data here are available on request.
